# Relapse according to antipsychotic treatment in schizophrenic patients: a propensity-adjusted analysis

**DOI:** 10.1186/1471-244X-11-24

**Published:** 2011-02-11

**Authors:** Aurelie Millier, Emmanuelle Sarlon, Jean-Michel Azorin, Laurent Boyer, Samuel Aballea, Pascal Auquier, Mondher Toumi

**Affiliations:** 1 Creativ-Ceutical France, rue du Faubourg Saint-Honoré, 75008 Paris, France; 2 National Institute of Health and Medical Research, INSERM, U669, Maison de Solenn, Boulevard de Port Royal, 75679 Paris, France; 3 University of Paris-Sud and University of Paris Descartes, UMR-S0669, 75014 Paris, France; 4 Department of Public Health, Hospital Center, Creil/Senlis, 60309 Senlis, France; 5 Department of Psychiatry, University Hospital Ste-Marguerite, Boulevard Sainte-Marguerite, 13009 Marseille, France; 6 Department of Public Health, EA 3279 Research Unit, University Hospital, Boulevard Jean Moulin 13385 Marseille, France; 7 UCBL 1 - Chair of Market Access University Claude Bernard Lyon I, Decision Sciences & Health Policy, Boulevard du 11 Novembre 1918, 69622 Villeurbanne, France

## Abstract

**Objective:**

To compare the rate of relapse as a function of antipsychotic treatment (monotherapy vs. polypharmacy) in schizophrenic patients over a 2-year period.

**Methods:**

Using data from a multicenter cohort study conducted in France, we performed a propensity-adjusted analysis to examine the association between the rate of relapse over a 2-year period and antipsychotic treatment (monotherapy vs. polypharmacy).

**Results:**

Our sample consisted in 183 patients; 50 patients (27.3%) had at least one period of relapse and 133 had no relapse (72.7%). Thirty-eight (37.7) percent of the patients received polypharmacy. The most severely ill patients were given polypharmacy: the age at onset of illness was lower in the polypharmacy group (p = 0.03). Patients that received polypharmacy also presented a higher general psychopathology PANSS subscore (p = 0.04) but no statistically significant difference was found in the PANSS total score or the PANSS positive or negative subscales. These patients were more likely to be given prescriptions for sedative drugs (p < 0.01) and antidepressant medications (p = 0.03). Relapse was found in 23.7% of patients given monotherapy and 33.3% given polypharmacy (p = 0.16). After stratification according to quintiles of the propensity score, which eliminated all significant differences for baseline characteristics, antipsychotic polypharmacy was not statistically associated with an increase of relapse: HR = 1.686 (0.812; 2.505).

**Conclusion:**

After propensity score adjustment, antipsychotic polypharmacy is not statistically associated to an increase of relapse. Future randomised studies are needed to assess the impact of antipsychotic polypharmacy in schizophrenia.

## 1. Background

Antipsychotic medication is described as the cornerstone of schizophrenia treatment, as it offers benefits for controlling symptoms and preventing relapse. Antipsychotic monotherapy is generally recommended by guidelines as the treatment of choice [1]. However, treatment resistance represents a significant clinical problem [2]. One-fifth to one-third of people with schizophrenia are considered to have illness that is resistant to treatment [3]. In this context, antipsychotic combination treatment, also called antipsychotic polypharmacy, has been frequently used in clinical practice. An increasing trend of antipsychotic polypharmacy has been described in Western countries [4], and the prevalence rate is estimated to be between 27% and 60% [5-9].

However, it remains unclear if there is an evidence base to support antipsychotic polypharmacy [3,6]. According to several observational studies, antipsychotic polypharmacy was associated with higher rates of extrapyramidal side effects than antipsychotic monotherapy. Further, antipsychotic polypharmacy was also reported to decrease adherence to treatment and to increase relapse and mortality compared to antipsychotic monotherapy [3,6,10,11]. However, in a recent meta-analysis Correll et al. [2] found that antipsychotic polypharmacy might be superior to monotherapy with respect to general measures of efficacy in certain clinical situations. Other studies did not find any statistical link between polypharmacy and mortality risk [12-14].

Consequently, the impact of antipsychotic polypharmacy requires further study to derive clinical recommendations [2]. According to Faries et al. [6], the reasons for the inconsistent findings may stem from methodological issues that need to be considered. Available randomised evidence is limited [2] because of the difficulties in conducting randomised controlled trials on this subject. Primarily, studies of an observational nature have been conducted and have provided useful information. However, using observational data to compare outcomes associated with antipsychotic polypharmacy may result in biased estimates [15]. Because the type of treatment (monotherapy or polypharmacy) was not randomly assigned in these prior studies, patients with specific characteristics, such as disease severity, could have been more likely to have been treated with polypharmacy. As these characteristics might be related to study outcomes, a direct comparison between patients with monotherapy and polypharmacy could have been biased. Moreover, the majority of studies with antipsychotics thus far have been of relatively short duration, and consequently there is a lack of evidence regarding their efficacy in the prevention of relapse in the long term [2].

The objective of the present study was to compare the occurrence of relapse according to antipsychotic treatment at baseline (monotherapy vs. polypharmacy), in a two-year observational cohort of French schizophrenic patients, using a propensity-adjusted analysis. Propensity analysis attempts to compare outcomes between two groups that have a similar distribution of measured covariates and, in this way, approximates the conditions of random site-of-treatment assignment [16].

## 2. Methods

### 2.1 Study design and sample

The data are from the European Schizophrenia Cohort (EuroSC), conducted in the UK, France, and Germany. A detailed description of the European Schizophrenia Cohort has been published earlier [17]. In brief, it is a naturalistic 2-year follow-up of a cohort of people suffering from schizophrenia. The principle objective of the EuroSC was to identify and describe the types of treatment and methods of care for people with schizophrenia and to correlate these with clinical outcomes, states of health, and quality of life [17-24]. In our study, we only included French samples to control for country variation in the management of schizophrenia, which can be a confounding bias. We have shown previously that service use varied considerably between the three participating countries [19]. The French health system may offer an interesting approach: universal access to care, totally free health-care, and access to the most appropriate treatment, regardless of cost. This cohort of people suffering from schizophrenia was from three catchment areas in France: northern France (Lille), central France (Lyon and Clermont-Ferrand), and southern France (Marseille and Toulon). Each of these areas covers an urban centre of approximately one million inhabitants living in a city or in medium-size towns. In each area, patients treated in the "psychiatric sector" [25] were identified according to the following criteria: diagnosis of schizophrenia according to the DSM-IV criteria [26], aged 18 to 64 years, and French as native language. Random sampling from these patients was used to generate a representative sample.

A total of 183 patients were followed for a 2-year period from 1998 to 2000, with data collected every 6 months. If the participant withdrew consent at any time or if the participant was lost to follow-up, data collected up to this point were used in analysis. This project was conducted in accordance with the Declaration of Helsinki and French Good Clinical Practices [27,28]. The protocol of this study was approved by the Institution Review Board or the Ethic Committee responsible for the participating hospital or institution. Written informed consent was obtained from each participant after the study details had been fully explained.

### 2.2 Data collection

The following data were collected.

1. Socio-demographic information: gender, age, and living situation.

2. Clinical characteristics: psychotic symptoms based on the Positive and Negative Syndrome Scale (PANSS), which comprises three different subscales (positive, negative and general psychopathology) [29,30]; Functioning based on the Global Assessment of Functioning (GAF) scale [31], the Global Assessment of Relational Functioning (GARF) [32] and the Social and Occupational Functioning Assessment Scale (SOFAS) [33]; depression based on the Calgary Depression Scale for Schizophrenia (CDSS), specifically designed for schizophrenic patients, which evaluates depression independent of extra-pyramidal and negative symptoms [34,35].

3. Drug information: antipsychotic medication (monotherapy vs. polypharmacy): at baseline, patients were queried about their use of medications. Monotherapy (polypharmacy) was defined as the occurrence of one (more than one) ongoing antipsychotic medication prescription on the day of the visit; sedative drugs, antidepressant and side-effects co-treatment; the Simpson and Angus Scale (SAS) [36], the Barnes Akathisia Scale (BAS) [37], and the Abnormal Involuntary Movement Scale (AIMS) [38] were used to assess the side-effects; the Rating of Medication Influences (ROMI) Scale was used to evaluate adherence to treatment [39]. The ROMI is a reliable and valid instrument that can be used to assess the patient's subjective reasons for medication compliance and non-compliance.

4. The number of previous hospitalisations.

5. Quality of life (QoL) questionnaire:

SF-36 is a generic, self-administered QoL questionnaire consisting of 36 items describing 8 dimensions: Physical Functioning (PF); Social Functioning (SF); Role--Physical Problems (RPP); Role--Emotional Problems (REP); Mental Health (MH); Vitality (VIT); Bodily Pain (BP); and General Health (GH). Each dimension is scored within a range from 0 (low QoL level) to 100 (high QoL level) [40,41].

### 2.3 Study outcome

Our primary outcome was relapse on a 2-year period, defined according to a usual, clinically reproducible and validated definition [42,43]: (1) hospitalisation due to worsening of psychotic symptoms or an unequivocal worsening of psychotic symptoms of such magnitude that hospitalisation appeared imminent, or (2) a re-emerge of florid psychotic symptoms such as delusions, hallucinations, bizarre behaviour, or (3) thought disorder lasting seven days or more.

This information was collected by routine clinical interview by a psychiatrist every six months, and relapse was defined using information regarding the baseline characteristics of the patient. Additional relevant information was obtained from the medical record and also through staff interviews.

### 2.4 Statistical analysis

Characteristics for patients were compared using the Chi-squared or Fisher exact tests for categorical variables and the Wilcoxon rank sum test for continuous variables. We performed a propensity score analysis to adjust for imbalances in baseline characteristics between patients with monotherapy and polypharmacy. For this purpose, we first developed a nonparsimonious logistic regression model to derive a propensity score for patients receiving polypharmacy based on all the covariates [44]. This logistic regression model yielded a c-statistic of 0.80, indicating a strong ability to discriminate between monotherapy and polypharmacy. This logistic regression model was used to estimate a propensity score for each patient, corresponding to the probability of being treated using polypharmacy. Patients were stratified by quintile of increasing propensity score. To validate our propensity score adjustment, we checked for adequate overlap in propensity scores for monotherapy and polypharmacy within each quintile and for the absence of significant residual imbalances in patient characteristics after adjustment for quintile of the propensity score. A multivariable Cox proportional hazards model was used to estimate the Hazard Ratio (HR) and its corresponding 95% confidence interval (CI) of relapse associated with polypharmacy after adjusting for the strata of propensity score.

Statistical analyses were carried out using SAS 9.1. The statistical significance level was set at p < 0.05 in a two-sided test.

## 3. Results

Our sample consisted of 183 patients; 50 patients (27.3%) had at least one relapse and, 133 patients had no relapse (72.7%). Males comprised 70.9% of patients, and the mean age was 24.8 years (standard deviation = 8.0). Of the 183 patients who were included in the study, 45 patients did not complete the two years of follow up (24.6%). The characteristics at baseline were similar for patients with complete follow up (n = 138) and without complete follow up (n = 45) (all p-values > 0.05).

### 3.1 Baseline characteristics of patients with monotherapy and polypharmacy

Characteristics for the monotherapy and polypharmacy groups are shown in Table 1. Sixty-two (62.3%) of the patients had monotherapy, and 37.7% of patients received polypharmacy. In the polypharmacy group, 54 patients received two antipsychotics (78.3%), and 15 patients received three antipsychotics (21.7%). Twenty-two patients in the polypharmacy group (31.9%) and 37 patients in the monotherapy group (32.5%) received a depot antipsychotic medication. There was no significant difference in socio-demographic and clinical characteristics between the two groups, except for the age at onset of illness, which was lower in the polypharmacy group (p = 0.03), and the general psychopathology PANSS score, which was higher in the polypharmacy group (p = 0.04). The proportion of patients receiving antidepressant and sedative drugs was significantly higher in the polypharmacy group (respectively p = 0.03 and p < 0.01). Side effects, as assessed with the AIMS, BAS, and SAS, did not differ between the two groups, whereas we identified a higher proportion of patients with drugs to correct side effects in the polypharmacy group (p < 0.01). Concerning QoL scores, we did not find any statistical difference except for the Role-Emotional limitations (problems with work or other daily activities as a result of emotional problems), which was lower in the polypharmacy group (p = 0.03).

**Table 1:** Baseline characteristics for schizophrenic patients with monotherapy and polypharmacy (n = 183).

	Patients with monotherapy (n = 114)		Patients with polypharmacy (n = 69)		*P *value
	M	(SD)^1^	M	(SD)	
**Socio-demographic characteristics**					
Gender (male), N (%)^2^	79	(69.3)	50	(73.5)	0.54
Age	37.9	(10.5)	40.6	(10.3)	0.08
Living conditions (Alone), N (%)	35	(30.7)	30	(43.5)	0.08

**Clinical characteristics**					
Age at onset of illness	25.43	(8.20)	22.65	(7.28)	**0.03**
Total PANSS^3 ^score	65.3	(18.0)	70.0	(22.2)	0.14
Positive PANSS score	14.0	(5.3)	13.7	(5.6)	0.74
Negative PANSS score	18.0	(6.9)	19.6	(8.2)	0.16
General Psychopathology PANSS score	33.3	(9.8)	36.7	(11.6)	**0.04**
GAF^4 ^score	54.0	(14.0)	51.0	(16.3)	0.20
GARF^5 ^score	55.7	(16.7)	54.3	(17.7)	0.59
SOFAS^6 ^score	53.9	(13.4)	50.7	(15.7)	0.15
CDSS^7 ^score	2.7	(3.3)	3.8	(4.1)	0.07

**Medication**					
Drugs for side-effects^§^, N (%)	40	(35.0)	42	(60.9)	**<0.01**
Sedative drugs^§§^, N (%)	49	(43.0)	46	(66.7)	**<0.01**
Antidepressant, N (%)	18	(15.8)	20	(29.0)	**0.03**
AIMS^8^	2.7	(4.3)	3.1	(4.6)	0.53
BAS^9^	1.0	(1.9)	1.1	(2.1)	0.71
SAS^10^	2.9	(3.3)	3.8	(4.0)	0.13
ROMI^11^					
Compliance score	12.2	(2.8)	13.0	(2.7)	0.09
Non compliance score	14.2	(3.7)	14.0	(3.8)	0.74

**Number of previous hospitalisations**	5.5	(5.3)	7.5	(6.9)	0.05

**Quality of life: SF-36**					
Physical Functioning	82.0	(20.5)	76.3	(22.9)	0.10
Role-Physical Limitations	74.2	(32.9)	65.9	(40.2)	0.15
Bodily Pain	72.7	(26.3)	71.9	(27.6)	0.85
General Health	58.2	(21.4)	58.3	(21.0)	0.98
Vitality	50.9	(19.1)	48.0	(17.9)	0.34
Mental Health	63.4	(20.4)	59.5	(17.3)	0.21
Role-Emotional Limitations	74.3	(36.4)	60.8	(40.2)	**0.03**
Social Functioning	68.9	(30.1)	67.1	(25.8)	0.70

**Relapse**, N (%)	27	(23.7)	23	(33.3)	0.16

^1 ^Mean (Standard Deviation)

^2 ^Effective (Percentage)

^3 ^Positive and Negative Syndrome Scale; ^4^Global Assessment Functioning; ^5^Global Assessment of Relational Functioning; ^6^Social and Occupational Functioning Assessment Scale; ^7^Calgary Depression Scale for Schizophrenia; ^8^Abnormal Involuntary Movement Score; ^9^Barnes Akathisia score; ^10^Simpson-Angus score; ^11^Rating of Medication Influences Scale.

§ Drugs for side effects: Biperiden hydrochloride, Tropatepine hydrochloride, and Trihexyphenidyl hydrochloride.

§§ Sedative drugs: Benzodiazepines, Antihistamines, and Hypnotics.

Values significant at the 5% level are marked in bold.

### 3.2 Factors associated with relapse

Characteristics for the patients with relapse and no relapse are shown in Table 2. Patients with relapse were significantly younger than patients with no relapse (p = 0.04). In the same way, the age at onset of illness was lower for patients with relapse than for those without relapse (p < 0.01). Patients with relapse were also less likely to report living independently (p = 0.04). Except for these three characteristics, no significant differences were found in the univariate analysis. The proportion of polypharmacy subjects between the relapse (46.0%) and non-relapse (34.6%) groups did not differ significantly (p = 0.15).

**Table 2:** Univariate and propensity score-stratified models: Hazard Ratio (HR) and its corresponding 95% confidence interval (CI) for risk factors associated with relapse (n = 183).

	Patients with relapse (n = 50)		Patients with no relapse (n = 133)			Propensity score-stratified model	
	**M**	**(SD)^1^**	**M**	**(SD)**	***P *value**	**HR**	**(95% CI)**

**Socio-demographic characteristics**							
Gender (male), N (%)^2^	34	(68.0)	95	(72.0)	0.60	-	-
Age	36.3	(9.7)	39.9	(10.6)	**0.04**	-	-
Living conditions (Alone), N (%)	12	(24.0)	53	(39.9)	**0.04**	-	-

**Clinical characteristics**							
Age at onset of illness	21.5	(5.6)	25.4	(8.4)	**<0.01**	-	-
Total PANSS^3 ^score	66.4	(18.7)	67.4	(20.2)	0.76	-	-
Positive PANSS score	14.6	(5.4)	13.6	(5.5)	0.26	-	-
Negative PANSS score	17.7	(6.5)	19.0	(7.8)	0.31	-	-
General Psychopathology PANSS score	34.0	(10.9)	34.8	(10.5)	0.66	-	-
GAF^4 ^score	51.9	(14.2)	53.3	(15.2)	0.58	-	-
GARF^5 ^score	54.2	(15.9)	55.5	(17.5)	0.64	-	-
SOFAS^6 ^score	52.0	(14.4)	52.9	(14.4)	0.72	-	-
CDSS^7 ^score	3.9	(4.5)	2.8	(3.3)	0.11	-	-

**Medication**							
Drugs for side-effects, N (%)	19	(38.0)	63	(47.4)	0.26	-	-
Sedatives drugs, N (%)	31	(62.0)	64	(48.1)	0.09	-	-
Antidepressants, N (%)	13	(26.0)	25	(18.8)	0.28	-	-
AIMS^8^	2.7	(4.3)	2.9	(4.4)	0.82	-	-
BAS^9^	1.1	(2.0)	1.0	(2.0)	0.74	-	-
SAS^10^	3.2	(3.1)	3.3	(3.8)	0.81	-	-
ROMI^11^						-	-
Compliance score	12.9	(2.6)	12.4	(2.8)	0.20		
Non compliance score	14.5	(3.8)	14.0	(3.7)	0.40		

**Number of previous hospitalisations**	7.7	(7.2)	5.7	(5.5)	0.10		

**Quality of life:SF-36**							
Physical Functioning	83.3	(18.8)	78.8	(22.3)	0.23	-	-
Role-Physical Limitations	66.5	(39.1)	72.7	(34.6)	0.32	-	-
Bodily Pain	69.4	(27.6)	73.4	(26.4)	0.39	-	-
General Health	55.8	(22.5)	59.1	(20.8)	0.38	-	-
Vitality	51.5	(16.6)	49.3	(29.4)	0.50	-	-
Mental Health	61.9	(19.5)	62.1	(19.4)	0.96	-	-
Role-Emotional Limitations	63.2	(39.1)	71.5	(38.0)	0.22	-	-
Social Functioning	68.6	(27.7)	68.2	(29.0)	0.93	-	-

**Polypharmacy**	23	(46.0)	46	(34.6)	0.15	1.686	(0.812; 2.505)

^1 ^Mean (Standard Deviation)

^2 ^Effective (Percentage)

^3 ^Positive and Negative Syndrome Scale; ^4^Global Assessment Functioning; ^5^Global Assessment of Relational Functioning; ^6^Social and Occupational Functioning Assessment Scale; ^7^Calgary Depression Scale for Schizophrenia; ^8^Abnormal Involuntary Movement Score; ^9^Barnes Akathisia score; ^10^Simpson-Angus score; ^11^Rating of Medication Influences Scale.

Values significant at the 5% level are marked in bold.

In the propensity-adjusted analysis, antipsychotic polypharmacy was not statistically associated with an increase of relapse: HR = 1.686 (0.812; 2.505).

## 4. Discussion

We found that antipsychotic polypharmacy in schizophrenic patients was not statistically associated with an increase in relapse in this observational study relying on a propensity score adjustment. Although a recent meta-analysis showed that antipsychotic polypharmacy may be superior to monotherapy [2], the majority of previous studies reported higher rates of side effects and relapse [3,6,10,11,45].

Several hypotheses can be proposed to explain our result.

One hypothesis is that previous studies have varied in design, potential predictors, sample examined, and the manner in which relapse was defined and measured [6]. Our study presents characteristics that can explain why our results are not entirely consistent with those of other studies. Our findings are based on naturalistic observational data. The results of observational studies reflect the patterns of practice and can be considered as more meaningful than clinical trials to evaluate effectiveness, notably on long-term outcomes such as relapse. We also conducted a 2-year follow up of schizophrenic patients, which is longer than those used in the majority of clinical trials and observational studies [3]. Clinical trials with antipsychotics thus far have been of relatively short duration [2]. Finally, we used a propensity score adjustment rarely performed on the previous observational studies. A propensity score is a better option than multivariate logistic regression when events are few and various confounding factors coexist [46].

Our findings suggest that the most severely ill patients were given polypharmacy, which can potentially bias findings of previous studies. In our study, the age at illness onset was lower in the polypharmacy group than in the monotherapy group. The age of illness onset is widely accepted as having particularly powerful clinical and prognostic significance. A recent meta-analysis supports the view that severity of disease process is associated with early onset [47]. Patients receiving polypharmacy also presented a higher general psychopathology PANSS score than patients with monotherapy. However, this statistically significant difference may not be clinically relevant, and no statistically significant difference was found in the PANSS total score or the PANSS positive or negative subscales. They were also more likely to be given prescriptions for sedative drugs than patients receiving monotherapy. These findings are consistent with a study showing that patients who received antipsychotic combinations exhibited more positive and excited symptoms than patients given prescriptions for monotherapy [48]. Additionally, the findings support clinicians' reports of using polypharmacy for refractory psychotic symptoms [49-51]. To a lesser extent, patients with polypharmacy were more likely to receive antidepressant medications. This result is concordant with a recent study showing that receiving antidepressants was significantly associated with receiving polypharmacy [52]. This association suggests that patients receiving polypharmacy present displayed more depressive symptoms than did patients receiving monotherapy, and studies have shown that schizophrenic patients with depressive symptoms have poorer long-term functional outcomes [53]. Consequently, the purported association between polypharmacy and poor outcome does not necessarily mean that polypharmacy leads to poor outcome; it may be that poor outcome may lead to aggressive prescription practices that includes high doses and polypharmacy [54]. This major confound, which is not systematically assessed, needs to be addressed in future studies.

Consistent with the results of previous reports [49,55], in our study, patients receiving polypharmacy were more likely to also receive drugs for side-effects than patients with monotherapy. Interestingly, we note in our study that the side effects were assessed with three different standardised instruments, and the adherence of the patients did not differ between the two groups. This can also explain our result by controlling the relation between polypharmacy, side effects as predictor of poor medication adherence, and relapse. Indeed, experts have endorsed side effects or a general fear of side effects as one important factor leading to adherence problems in schizophrenia [56]. In the same way, experts have rated persistent positive or negative symptoms in schizophrenia as the most important symptomatic contributors to adherence problems [56]. The combination of polypharmacy and sedative drugs can explain the absence of differences in persistent positive or negative symptoms between monotherapy and polypharmacy groups. Consequently, this may explain that antipsychotic polypharmacy was not associated to an increase of relapse.

### Perspectives and limitations

Our study had several limitations.

First, the treatment (monotherapy or polypharmacy) was not based on random assignment; therefore, the results may be confounded by other factors. Although the propensity score can adjust for confounding by indication and selection bias, we cannot eliminate residual confounding due to unobserved factors [57]. This limitation of the current work can be moderated by the broad spectrum of characteristics collected in our study. However, well-designed randomised controlled trials are needed to determine the impact of polypharmacy on relapse in schizophrenia.

Second, several interesting data were not analysed. Information regarding dosages of different antipsychotic was not available. Moreover, antipsychotic combinations can be considered as a heterogeneous group, and the different combinations might be associated with different risks of relapse. However, our sample was not sufficient to determine the effect of specific polytherapy combinations.

Third, our sample may not be representative of schizophrenic patients in all of France and in other countries. However, the proportion of polypharmacy and relapse that are in line with previous studies [3,5,6,8,9,14] may indicate a good representativeness.

Fourth, it is possible that our study lacked statistical power to detect a difference in the rate of relapse. However, our analytical sample comprised 183 patients, 50 of whom had a relapse. This sample size was larger than that of several published randomised controlled trials designed to test the efficacy of polypharmacy vs. monotherapy, especially when we consider the length of the follow up, which were usually shorter in these prior studies than in our study [2].

Finally, a major methodological problem remains in the definition of relapse for schizophrenic patients and the definition of monotherapy and polypharmacy. There are no generally accepted criteria for relapse [42]. However, we have chosen the most consensual definition in the recent scientific literature [42,43,58]. Methodological issues to be addressed in future trials should include clinically relevant relapse criteria. In the same way, the definitions of monotherapy and polypharmacy are not consensual. We defined the exposure treatment groups at baseline in a standard way: receiving one antipsychotic at baseline, regardless of medication class or molecule, compared with receiving two or more antipsychotics. We assumed that exposure was relatively stable over time and that the occurrence of relapse may be related to the exposure treatment at baseline. The clinical significance of this study should be cautiously interpreted in accordance with this chosen definition.

## 5. Conclusion

In conclusion, antipsychotic polypharmacy is not statistically associated with an increase in relapse after the propensity adjustment is made. Well-designed randomised controlled trials are needed to assess the impact of antipsychotic polypharmacy in schizophrenia.

## Role of Funding Source

The study was funded by H. Lundbeck A/S.

## Authors' contributions

AM, ES and LB wrote the manuscript.

All authors designed the study and wrote the protocol.

AM, ES and LB conducted the literature searches and analyses.

AM conducted the statistical analyses.

All authors contributed to and approved the final manuscript.

## Declaration of interest

The authors declare that they have no competing interests.

## Pre-publication history

The pre-publication history for this paper can be accessed here:

http://www.biomedcentral.com/1471-244X/11/24/prepub
